# miR-23b/SP1/c-myc forms a feed-forward loop supporting multiple myeloma cell growth

**DOI:** 10.1038/bcj.2015.106

**Published:** 2016-01-15

**Authors:** M Fulciniti, N Amodio, R L Bandi, A Cagnetta, M K Samur, C Acharya, R Prabhala, P D'Aquila, D Bellizzi, G Passarino, S Adamia, A Neri, Z R Hunter, S P Treon, K C Anderson, P Tassone, N C Munshi

**Affiliations:** 1 Dana-Farber Cancer Institute, Harvard Medical School, Boston, MA, USA; 2 Department of Experimental and Clinical Medicine, Magna Græcia University of Catanzaro, Salvatore Venuta Campus, Catanzaro, Italy; 3 VA Boston Healthcare System, Harvard Medical School, Boston, MA, USA; 4 Department of Biology, Ecology and Earth Science (DiBEST), University of Calabria, Arcavacata di Rende, Cosenza, Italy; 5 Department of Medical Sciences, University of Milan, Hematology 1, IRCCS Policlinico Foundation, Milan, Italy

## Abstract

Deregulated microRNA (miR)/transcription factor (TF)-based networks represent a hallmark of cancer. We report here a novel c-Myc/miR-23b/Sp1 feed-forward loop with a critical role in multiple myeloma (MM) and Waldenstrom's macroglobulinemia (WM) cell growth and survival. We have found miR-23b to be downregulated in MM and WM cells especially in the presence of components of the tumor bone marrow milieu. Promoter methylation is one mechanism of miR-23b suppression in myeloma. In gain-of-function studies using miR-23b mimics-transfected or in miR-23b-stably expressing MM and WM cell lines, we observed a significant decrease in cell proliferation and survival, along with induction of caspase-3/7 activity over time, thus supporting a tumor suppressor role for miR-23b. At the molecular level, miR-23b targeted Sp1 3′UTR and significantly reduced Sp1-driven nuclear factor-κB activity. Finally, c-Myc, an important oncogenic transcription factor known to stimulate MM cell proliferation, transcriptionally repressed miR-23b. Thus *MYC*-dependent miR-23b repression in myeloma cells may promote activation of oncogenic Sp1-mediated signaling, representing the first feed-forward loop with critical growth and survival role in myeloma.

## Introduction

The majority of oncogenic signaling pathways converge on sets of transcription factors (TFs) that ultimately control gene expression patterns involved in tumor formation and progression.^1^ Consequently, TF genes have been linked to cancer pathogenesis and drug resistance, making them attractive targets for therapy.^2^ In multiple myeloma (MM), a wide deregulation of the transcriptional machinery leading to alteration in cell cycle pathways has been recently described by our group in clinically annotated patient subgroups.^3^ Moreover, we have demonstrated increased activity of the TF Sp1, a ubiquitous zinc-finger TF that binds guanine–cytosine-rich elements in the promoter region of inducible genes,^4, 5^ in MM^7,6^ and Waldeström's macroglobulinemia (WM).^8^ In these malignancies, Sp1 was found to trigger the nuclear factor-κB (NF-kB) pathway, which sustains proliferation, survival and drug resistance in tumor cells. Importantly, genetic as well as pharmacological targeting of Sp1 was able to reduce tumor growth both *in vitro* and *in vivo,*^6, 8^ thus indicating that Sp1 is a suitable target for therapeutic intervention in MM and WM. Expression of TFs is modulated by a number of genomic and epigenomic changes as well as various transcriptome modifiers. Among such transcriptome modifiers there are microRNAs (miRNAs), small non-coding RNAs (20–22 nucleotides in length) that play an important role in regulation of gene expression, virtually affecting all cellular processes such as differentiation, proliferation, survival and apoptosis.^9, 10^ Genetic disorders and complex diseases have been found to be associated with perturbations of the intertwined regulatory network between TFs and miRNAs.^11^ Moreover, TFs and miRNAs frequently form feed-forward loops to regulate transcription of functionally critical genes.^12^

Over the last decade, a wealth of data has emerged supporting a role for miRNAs in MM^14,13^ and WM pathobiology,^15, 16^ acting as tumor suppressor miRNAs or onco-miRNAs. In this study, we have identified miR-23b as a negative regulator of Sp1 expression and provided evidence of the tumor suppressor role of miR-23b, and its downregulation as a consequence of epigenetic mechanisms, supporting the existence of a novel feed-forward loop with a critical growth and survival role in MM and WM.

## Materials and methods

### Cells

MM and WM cell lines were cultured in RPMI-1640 containing 10% fetal bovine serum, 2 mmol/l l-glutamine, 100 U/ml penicillin and 100 mg/ml streptomycin. They were kindly provided by sources previously described,^8^ ATCC (Manassas, VA, USA) or the German Collection of Microorganisms and Cell Cultures (Braunschweig, Germany). Primary WM and MM cells from bone marrow aspirates from among patients following informed consent and Dana-Farber Cancer Institute IRB approval were isolated using Ficoll-Hypaque density gradient sedimentation. Primary CD138^+^ MM cells from patients were purified using positive selection with CD138-microbeads (MiltenyiBiotec, San Diego, CA, USA). Primary WM cells were obtained using CD19 micro-bead selection (MiltenyiBiotec). Cell purity (more than 90%) was confirmed by flow cytometric analysis. Residual CD138^−^ bone marrow mononuclear cells were cultured for 3–6 weeks to generate bone marrow stromal cells. Peripheral blood mononuclear cells were obtained either by Ficoll-Hypaque density gradient sedimentation from healthy subjects or from patients.

### miR23b profiling in MM patients

miRNA expression levels were obtained by data set GSE37053^17^ and GSE17498^18^ or by TaqMan miRNA assays (Applied Biosystems, Carlsbad, CA, USA).

### Luciferase reporter experiments

The pGL3 Luciferase plasmid containing the NF-κB-responsive elements cloned upstream of the firefly luciferase reporter gene was previously described.^8^ Transfections were performed by electroporation with the Neon transfection system (Life Technologies, Grand Island, NY, USA) at the previously reported electroporation settings,^19^ and renilla luciferase from pCMV-RL was included to normalize expression of firefly luciferase. The 3′UTR segment containing the target sites for miR-23b from the Sp1 gene (nucleotides 2503–5200) and a deletion mutant lacking nucleotides from 3774 to 3781 corresponding to the predicted miR-23b target site were cloned in pEZX-MT01 vector were purchased from Genecopoeia (Rockville, MD, USA). In all, 2.5 μg of the firefly luciferase reporter and 0.5 μg of pCMV-RL were used; for each plate, 200 nm of the synthetic miR-23b or miR-NC was used. Firefly and Renilla luciferase activities were measured consecutively using the dual-luciferase assay kit (Promega Corporation, Madison, WI, USA) as previously described.^20^ Data are expressed as luminescence from firefly luciferase divided by luminescence from renilla luciferase.

### Generation of stable cell lines

To generate cells stably expressing miR-23b, NCI-H929 and MWCL1 cells were transduced with V-(mir-cnt) or V-(mir-23b) lentiparticles according to the protocol from the manufacturer (Biosettia, San Diego, CA, USA). Efficiency of overexpression was validated by real-time PCR. Total RNA was extracted 72 h post-tranduction using mirVana miRNA Isolation Kit (Ambion, Thermo Fisher Scientific), followed by TaqMan miRNA assays (Applied Biosystems, Assay ID 000400). miR-23b expression was normalized on RNU44 (Applied Biosystems, Assay ID 001094). The relative level of miR-23b expression was determined by comparing with the negative control; V-(mir-cnt).myc precision LentiORF viral particles (Thermo Scientific, Lafayette, CO, USA) were used to overexpress myc in U266 MM cells.

### Transient transfection of miR-23b mimics and c-myc siRNA

Synthetic pre-miRNAs were purchased from Ambion (Applied Biosystems). Transfections were performed by electroporation with Neon transfection system (Life Technologies).^19, 21^ RNA interference for c-myc was carried out by using the TranSilent Human myc small interfering RNA (siRNA) (Panomics, Inc., Redwood, CA, USA). Non-targeting scrambled negative control siRNA (Panomics, Inc.) was used as a negative control.

### Immunoblotting

Western blotting was performed according to previously described protocols,^19^ to delineate expression levels of total protein (Sp1, survivin, caspase-3, caspase-8, NF-κB, ERK) and phospho-specific isoforms of NF-κB and ERK. Glyceraldehyde-3-phosphate dehydrogenase was used as a loading control (Santa Cruz Biotechnology, St Louis, MO, USA).

### Cell proliferation and viability assay

Cell proliferation was measured by [^3^H]thymidine (Perkin-Elmer, Boston, MA, USA) incorporation assay.^6^ Cell viability was analyzed by Cell-Titer Glow (CTG) (Promega, Madison, WI, USA). Study of caspase activity was performed using Caspases 3–7, -8, -9 Glow assays (Promega).

### Methylcellulose colony formation assays

V-(mir-cnt) or V-(mir-23b) cells were suspended in Iscove's modified Dulbecco's medium with 50% fetal bovine serum and plated on methylcellulose-based media (HSC004; R&D Systems, Minneapolis, MN, USA) containing 1.4% methylcellulose in Iscove's modified Dulbecco's medium, 25% fetal bovine serum, 2% bovine serum albumin, 2 mml-glutamine, 5 × 10^−5^ m 2-mercaptoethanol, 20 ng/ml recombinant human granulocyte macrophage colony-stimulating factor, 20 ng/ml recombinant human interleukin (IL)-3 and 50 ng/ml recombinant human SCF. Each condition was evaluated twice in triplicates. Colonies, defined as aggregates ⩾50 cells, were scored with an inverted microscope after 21 days incubation at 37 °C in a fully humidified atmosphere with 5% CO_2_.

### Cloning of miR-23b promoter region

We cloned the promoter region of miR-23b (chromosome location: chr 9 94725317-94726771), described in Zhou *et al.*,^22^ into pGL3 basic vector. The 1455-bp region containing the miR-23b promoter was PCR amplified from human genomic DNA by using primers containing a 6 -bp cloning adaptor with the SacI and *Xho*I restriction sites (Forward: 5′-GAGCTCGCAGCTAGCAGGGTGATG-3′ Reverse: 5′-CTCGAGACAGGGAGCGAA ACAGGGAGCGAACAGGTTA-3′). PCR was carried out in 100 μl containing EpiTaq PCR Buffer 1X, 0.3 mm dNTPs, 0.3 μm of each primers, 2.5 mm MgCl_2_, and PCR Enzyme 0.05 U (TaKaRa Bio, Clontech Laboratories, Mountain View, CA, USA). The PCR reaction consisted of 30 cycles (94 °C for 15 s for the denaturation step, 62 °C for 30 s for the annealing step and 72 °C for 2 min for the extension step). A denaturation step of 2 min at 94 °C preceded the first cycle while the last was followed by an extension step of 5 min at 72 °C. The resulting fragment was purified by DNA Clean & Concentrator TM-5 kit (Zymo Research, Irvine, CA, USA) and digested with SacI and *Xho*I enzymes (Promega) as recommended by the manufacturer. Then, the fragment was purified by DNA Clean & Concentrator TM- 5 kit (Zymo Research) and inserted by using T4 DNA ligase (Promega) into the SacI–*Xho*I sites upstream of the firefly luciferase reporter gene in the linearized pGL3-Basic vector (Promega). DNA construct was transformed into Top10 *Escherichia coli* cells by electroporation according to the standard protocols. The pGL3/miR-23b promoter plasmid was purified using ZR Plasmid Miniprep Classic (Zymo Research) and analyzed by automated sequencing in ABI PRISM 310 with the BigDye Terminator Cycle Sequencing Ready Reaction Kit (Applied Biosystems) to confirm that the sequence matched the original genomic sequences without PCR-generated errors.

### Quantitative DNA methylation analysis

Primers for quantitative DNA methylation experiments were designed by using EpiDesigner, a specialized tool for Sequenom'sEpiTYPER technology. A T7-promoter tag (CAGTAATACG ACTCACTATAGGGAGAAGGCT) was added to the reverse primer and a 10-mer tag sequence (AGGAAGAGAG) was added to the forward primer to balance the PCR primer length. Primer sequences are reported in Supplementary Table S1.

### Bisulfite treatment

Bisulfite conversion of DNA samples was performed by using EZ-96 DNA Methylation-Gold Kit (Zymo Research), as previously described.^23^

### PCR conditions and Sequenom EpiTYPER technology

The PCR reactions were performed in a total volume of 5 μl using 1 μl of bisulfite-treated DNA, EpiTaq PCR Buffer 1 ×, 0.4 μm of each primer, 0.3 mm dNTP mixture, 2.5 mm of MgCl_2_, 0.005 U TaKaRa EpiTaq HS (TaKaRa Bio, Clontech Laboratories). The thermal profile used for the reaction included a 4 min heat activation of the enzyme at 95 °C, followed by 40 cycles of denaturation at 94 °C for 20 s, annealing (for temperature see Supplementary Table S1) for 30 s, extension at 72 °C for 1 min, then 1 cycle at 72 °C for 3 min. In all, 0.5 μl of each PCR product was electrophoresed on 1.5% agarose gel to confirm successful PCR amplification and amplification specificity. Unincorporated dNTPs in the amplification products were dephosphorylated by adding 1.7 μl DNase-free water and 0.3 μl (0.5 U) shrimp alkaline phosphatase (Sequenom, Inc., San Diego, CA, USA). Each reaction was incubated at 37 °C for 40 min and shrimp alkaline phosphatase was then heat inactivated for 5 min at 85 °C. Subsequently, samples were incubated for 3 h at 37 °C with 5 μl of T-Cleavage reaction mix (Sequenom) containing 3.21 μl RNase-free water, 0.89 μl 5 × T7 polymerase buffer, 0.22 μl T cleavage mix, 0.22 μl 100 mm dithiothreitol, 0.40 μl T7 RNA, and DNA polymerase and 0.06 μl RNase A, for concurrent *in vitro* transcription and base-specific cleavage. The samples of cleaved fragments were then diluted with 20 μl water. Conditioning of the cleavage reaction was performed by adding 6 mg of clean resin. Ten nanoliters of the resultant cleavage reactions were spotted onto silicon matrix-preloaded chips (Spectro-CHIP; Sequenom) using a MassARRAY nanodispenser (Sequenom), and analyzed using the MassARRAY Compact System matrix-assisted laser desorption/ionization-time-of-flight mass spectrometer (Sequenom). The spectra's methylation ratios were calculated using EpiTYPER software v1.0 (Sequenom). The method yields quantitative results for each of the sequence-defined analytic units, referred to as CpG units, which contain either one individual CpG site or an aggregate of downstream CpG sites. Triplicate independent analyses from sodium bisulfite-treated DNA sample were undertaken. The effectiveness of the entire experimental procedure was also assayed by analyzing CpGenome Universal Unmethylated DNA (Chemicon International, Temecula, CA, USA) and CpGenome Universal Methylated DNA (Chemicon International) as control. Poor-quality and non-valuable data for the quantitative methylation of each CpG unit measured by a matrix-assisted laser desorption/ionization-time-of-flight mass spectrometer were excluded.

### *In vivo* study

V-(mir-cnt) or V-(mir-23b) H929 cells were injected s.c. in SCID mice. Tumor growth was measured in two perpendicular dimensions once every 3 days using a caliper and the following formula: *V=(a*^*2*^*x b)/2*; where *a* is the width of the tumor (smaller diameter) and *b* is the length (larger diameter).

### Statistical analysis

Data were analyzed using unpaired Student's *t-*tests comparing two conditions or a one-way analysis of variance with Bonferroni or Newman–Keuls correction for multiple comparisons using the Graphpad software. *P* <0.05 was considered significant. Statistical analyses for methylation experiments were performed using SPSS 15.0 statistical software (SPSS Inc., Chicago, IL, USA). The difference in the methylation rates of each CpG units as well as in global DNA methylation analysis between untreated and treated samples was analyzed by one-way analysis of variance and Student's *t-*test, with a significance level defined as *α*=0.05. Data are presented as means, and error bars in the figures depict standard deviation (s.d.).

## Results

### miR-23b represses TF SP1 expression and activity

**Figure 1:**
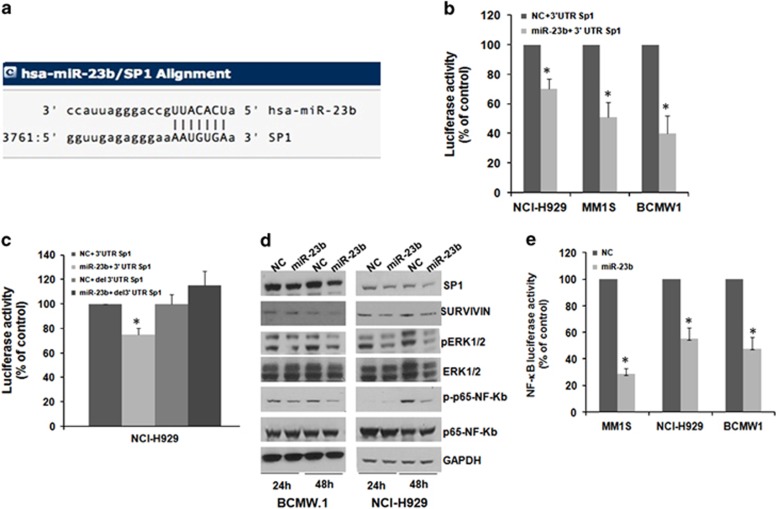
Sp1 targeting by miR-23b in MM and WM tumor cells. (**a**) Identification of a miR-23b-target sequence within Sp1 3′UTR by microRNA.org *in silico* target prediction. (**b**) Dual-luciferase assay of NCI-H929, MM1s and BCMW1 cells co-transfected with firefly luciferase constructs containing a portion of the 3′UTR of Sp1 (nts 2503–5200) and 200 nm of miR-23b or scrambled oligonucleotides (NC). The firefly luciferase activity was normalized to renilla luciferase activity. The data are shown as relative luciferase activity of miR-23b-transfected cells as compared with the control (NC) of a total of six experiments from three independent transfections. *P<0.01. (**c**) Dual-luciferase assay of NCI-H929 cells co-transfected with firefly luciferase constructs containing the 3′UTR of Sp1 or a deletion mutant lacking the predicted miR-23b target site (3′UTR del) and 200 nm of miR-23b or scrambled oligonucleotides (NC) as indicated. The firefly luciferase activity was normalized to renilla luciferase activity. *P<0.01. (**d**) Immunoblot analysis of Sp1, survivin, phosphorylated ERK1/2, phosphorylated p65, p65 in NCI-H929 and BCMW1 cells transfected with 200 nm miR-23b mimics or NC. GAPDH was used as a loading control. (e) Luciferase assay of MM1s, NCI-H929 and BCMW1 cells transfected with 200 nm of miR-23b mimics or NC and a firefly luciferase vector carrying the NF-κB-responsive elements. Renilla luciferase from pCMV-RL was used for normalization. Data are expressed as the ratio of firefly and renilla luciferase and are the mean of three different experiments performed in triplicate. *P<0.01. Abbreviation: GAPDH, glyceraldehyde-3-phosphate dehydrogenase.

Using cancer miRNA Transcriptome PCR Array (SureFIND) we first identified miR-23b as one of the most significant negative regulator of Sp1 expression (data not shown). Moreover, *in silico* search for target prediction (www.microRNA.org) indicates that Sp1 is a *bona fide* target of miR-23b (Figure 1a). To validate 3′UTR targeting by miR-23b, NCI-H929, MM1s and BCMW1 cells were co-transfected with synthetic miR-23b or scrambled mimics (NC), together with an expression vector carrying the 3′UTR of Sp1 cloned downstream of the luciferase reporter gene. A significantly lower luciferase activity in cells transfected with miR-23b mimics as compared with control was detected (Figure 1b). To confirm that this inhibitory activity was dependent on the binding of miR-23b to the predicted sequence, NCI-H929 cells were transfected with NC or miR-23b mimics along with the plasmid carrying the *wild-type* 3′UTR of Sp1 or a deletion mutant devoid of the miR-23b target sequence. As expected, the decrease in luciferase activity by miR-23b in cells transfected with the *wild-type* 3′UTR of Sp1 was no longer observed after deletion of the miR-23b target site (Figure 1c). Consistently, miR-23b mimics transfection in BCMW1 and NCI-H929 cells reduced Sp1 protein expression as well as the levels of known Sp1 transcriptional targets such as survivin, as assessed by immunoblotting analysis; a decrease in phosphorylated p65 (RELA) and MAPK (ERK1/2) was also observed after miR-23b overexpression (Figure 1d). Moreover, miR-23b transfection was able to reduce the activity of NF-κB-responsive elements in luciferase reporter assays (Figure 1e).

### miR-23b is dowregulated MM and WM tumor cells

**Figure 2:**
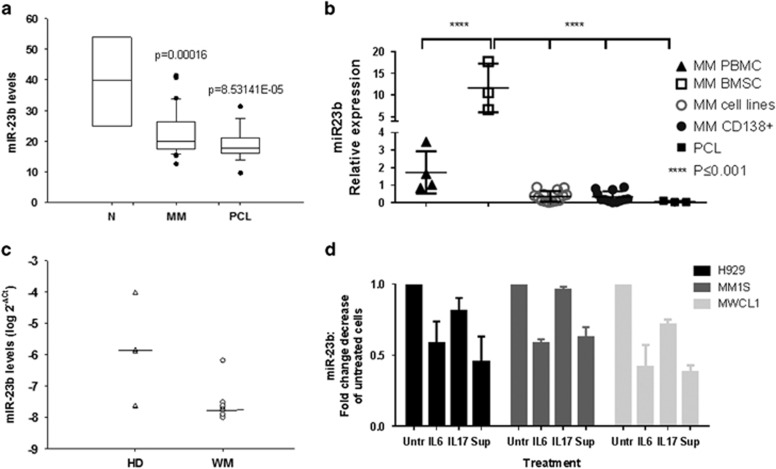
Relative expression of miR-23b in MM and WM tumor cells. (**a**) Box plot representation of mir-23b expression using Affimetrix Platform in a cohort of 38 multiple myeloma (MM) and 18 plasma cell leukemia (PCL) patients compared with normal PCs. (**b**) qRT–PCR analysis of miR-23b expression in a panel of MM cell lines (*n*=15), peripheral blood mononuclear cell (*n*=4) and BMSCs (*n*=3) from MM patients, CD138^+^ from MM patients (*n*=12) and PCL patients (*n*=3). (**c**) Expression of miR-23b in WM primary cells compared with normal CD19^+^ cells. (**d**) MM and WM cell lines were treated either with 10 ng/ml of IL-6, 100 ng/ml IL-17 or supernatant from tumor-derived BMSC suppressed the expression of miR-23b for 24 h. miR-23b expression was assessed by qRT–PCR using RNU44 as a loading control. Abbreviations: BMSC, bone marrow stromal cell; qRT–PCR, quantitative reverse transcriptase–PCR.

We examined miR-23b expression (Affymetrix, Santa Clara, CA, USA) in CD138^+^ myeloma cells from 38 MM patients and 18 plasma cell leukemia patients and found it to be downregulated compared with normal PCs (Figure 2a). The downregulation of miR-23b expression was also observed in several MM cell lines when compared with peripheral blood mononuclear cells and bone marrow stromal cells from MM patients, and in additional independent patient data set by quantitative PCR (Figure 2b). Interestingly, we have observed lower expression of miR-23b in WM, an indolent lymphoproliferative disease with high Sp1 activity^8^ (Figure 2c). Importantly we observed further downregulation of miR-23b expression in MM cells following interaction with bone marrow milieu. Treatment with both IL-6 or supernatant from MM-derived bone marrow stromal cell suppressed the expression of miR-23b (Figure 2d) in a time- and dose-dependent pattern (data not shown). Moreover, miR-23b is commonly repressed in autoimmune conditions by IL-17 (ref. 24), a cytokine shown to promote myeloma cell growth and inhibit its immune function. We have indeed observed a further decrease in miR-23b expression in MM cells after IL-17 treatment for 24 h. Our data indicate that the human bone marrow microenvironment modulates miR-23b levels in both MM and WM cells.

### miR-23b is silenced by promoter hypermethylation in MM

**Figure 3:**
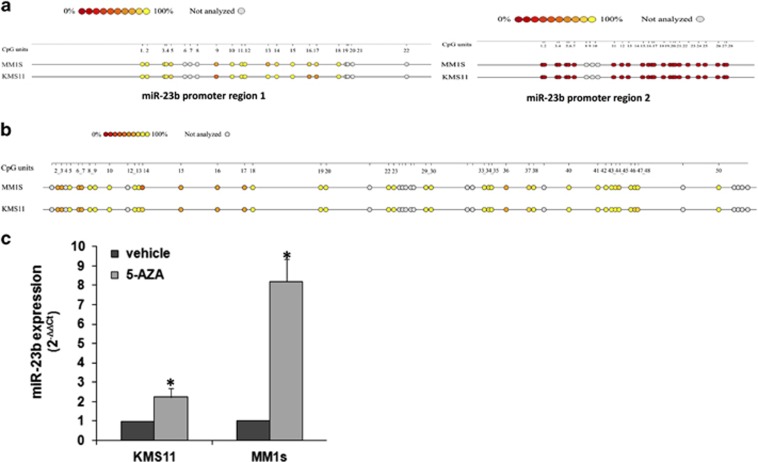
miR-23b promoter is hypermethylated in MM cells. Sequenom's EpiTYPER epigram panel representative of the methylation status (**a**) of the two CpG islands located within the promoter region of miR-23b or of (**b**) a 1536 bp region located 843 bp upstream and 597 bp downstream of miR-23b. Methylation data are displayed as color-filled circle and the color spectrum indicates the range of methylation of each CpG. Empty gray circles correspond to CpG sites that failed analysis. Data are reported as mean of three independents experiments. (**c**) Quantitative reverse transcriptase–polymerase chain reaction analysis for miR-23b expression in KMS11 and MM1S cells treated with 1 μm 5-azacytidine (5-AZA) for 48 h. *P<0.01.

Previous studies indicate that miR-23b is silenced in prostate cancer^25^ and glioblastoma^26^ as a consequence of promoter hypermethylation. We identified two CpG islands within the miR-23b promoter (CpG promoter region 1 and CpG promoter region 2, chr 9: 94725607-94726091 and 94726477-94726771, respectively), which were then analyzed for their methylation status in MM cells. As shown in Figure 3a, both KMS11 and MM1s cells showed a high degree of methylation of CpGs within the CpG promoter region 1. We also analyzed the methylation status of a 1536 bp CpG-rich region located 843 bp upstream and 597 bp downstream of miR-23b (chromosome location: chr 9: 95084365-95085901; Ensemble Genome Browser, Cambridge, UK), and observed majority of the CpG sites methylated (Figure 3b). The percentage of methylation of each CpG within the regions analyzed is reported in Supplementary Figure S1. Importantly, treatment of KMS11 and MM1s cells with the demethylating compound 5-azacytidine resulted in upregulation of miR-23b levels, as assessed by quantitative reverse transcriptase–PCR, indicating that miR-23b promoter hypermethylation may contribute to observed low miR-23b expression in MM (Figure 3c).

#### miR-23b overexpression suppresses cancer cell proliferation and caspase-3 activation *in vitro* and *in vivo*

**Figure 4:**
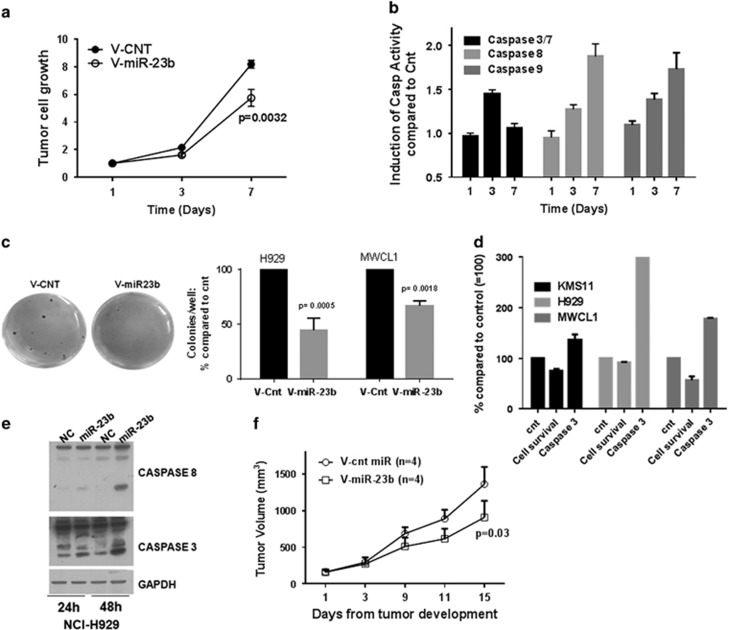
Functional role of miR23b replacement in MM and WM cells. (**a**) Cell growth of NCI-H929 myeloma cells stably expressing miR-23b (V-miR-23b) or control (V-CNT) was evaluated by [^3^H]Thymidine uptake at the indicated time. Data are presented as cell growth increase compared with Day 1. (**b**) Activation of Caspase-3, -8 and -9 was assessed by luminescence assay. (**c**) To test effects of miR-23b overexpression on the malignant phenotype of MM and WM cells, we measured colony formation in semisolid, methylcellulose media. Representative phase contrast images for H929 colonies formed in semisolid methylcellulose medium at day 21 for V-miR-23b and V-CNT cells are shown in the left panel. In the right panel, graphs depict average colony numbers (mean±s.d.) for NCI-H929 and MWCL1 V-miR-23b and V-CNT cells in methyl cellulose medium at day 21. (**d**) Transient transfection of miR-23b inhibited cancer cell survival as evaluated by cell titer glow assay, and induced caspase-3 activation as evaluated by luminescence assay. Data are presented as % of control cells. (**e**) WB analysis confirmed cleavage of caspase-3 and -8 in cells transiently transfected with miR-23b mimics compared with control cells. (**f**) Growth curve assess tumor size after subcutaneous injection of NCI-H929 myeloma cells transduced with miR-23b or scrambled control virus into the right posterior flank region of SCID mice. Data are shown as mean values±s.d.

We further assessed the functional significance of miR-23b in MM and WM cells by gain of function studies. A significant decrease in cell proliferation along with induction of caspase-3/7 activity was observed over time in MM and WM cell lines stably expressing miR-23b compared with control cells (Figures 4a and b). MiR-23b-transfected cells also had low colony formation ability, as the number of foci in miR-23b-expressing cells was decreased when compared with cnt-miR-transfected cells (Figure 4c). These data were confirmed using transient transfection with miR-23b mimics (Figures 4d and e). Finally, overexpression of miR-23b resulted in reduced tumor growth *in vivo* in SCID mice over the course of 2 weeks when compared with control cells (Figure 4f). These results indicate a tumor growth suppressor role for miR-23b in MM and WM.

#### c-myc/Sp1 dependent regulation of miR-23b expression

**Figure 5:**
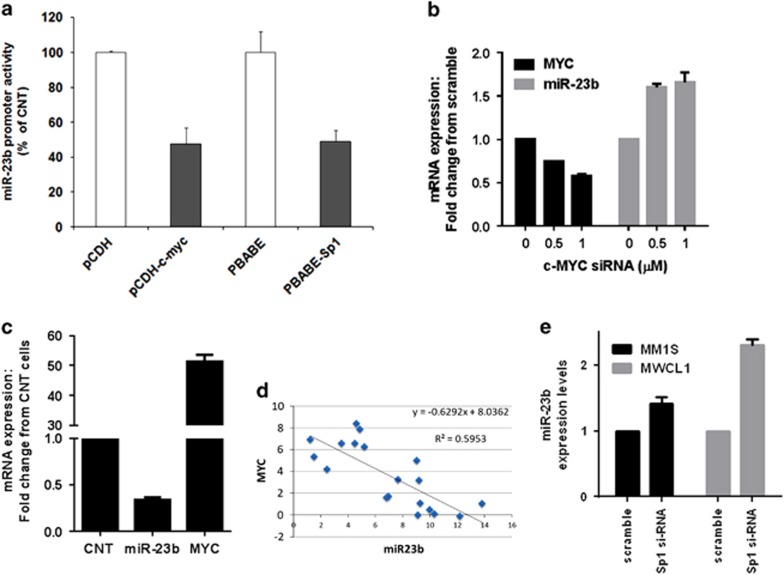
Transcriptional repression of miR-23b by Myc. (**a**) U266 cells were transfected with a firefly luciferase construct containing the miR-23b promoter, together with an Sp1 or c-myc expression constructs or the corresponding empty vectors; pCMV-RL was used for normalizing firefly luciferase activity. Results are expressed as the ratio between firefly and renilla luciferase of three independent experiments performed in triplicate. *P<0.01. (**b**) MM1S cells transfected using different concentrations of myc siRNA or scramble siRNA (Scr). Quantitative PCR (qPCR) analyses confirmed reduction in c-myc mRNA levels and increased miR-23b levels following transient transfection of MM1S cells with c-myc siRNA compared with cells transfected with control scrambled siRNA. (**c**) Stable c-myc overexpression was achieved in U266 cell line using Precision LentiORFs GFP-tagged. qPCR analyses confirmed increased in c-myc mRNA levels and decreased miR-23b levels following myc overexpression in U266. (**d**) Inverse correlation between mRNA levels of c-myc and miR-23b was evaluated in a cohort of 12 MM patients by qPCR, Pearson correlation and linear regression analysis. *R*=regression coefficient. (**e**) The effect of Sp1 knockdown on miR-23b in MM1S and MWCL1 cells transfected with Sp1 or control siRNA was assessed by qPCR and presented as change relative to control cells.

To shed light on the transcriptional regulation of miR-23b, we analyzed miR-23b promoter regions and identified several putative binding sites for oncogenic TFs such as c-myc, NF-kB and Sp1 (Supplementary Fig. 2). c-Myc, a known myeloma oncogenic TF, has been already shown to transcriptionally repress miR-23a and miR-23b in human prostate cancer cells;^27^ conversely, regulation of miR-23b by Sp1 has not been previously reported. To evaluate the effect of c-myc or Sp1 on miR-23b promoter transactivation, we transfected U266 cells with a plasmid carrying the miR-23b promoter cloned upstream of the luciferase reporter gene, along with expression vectors encoding for c-myc or Sp1. Luciferase assay demonstrated that c-myc or Sp1 overexpression reduced miR-23b promoter activity, thus suggesting that both TFs negatively regulate miR-23b expression (Figure 5a). To confirm transcriptional control of miR-23b by c-myc in MM, we evaluated its expression level in cells either silenced or overexpressing c-myc. Upon silencing of myc with specific siRNAs, we observed increased miR-23b expression in a dose-dependent way (Figure 5b). On the other hand, upregulation of c-myc in the c-myc-negative U266 cell line decreased miR-23b expression (Figure 5c). Importantly, we observed an inverse correlation between c-myc and miR-23b levels in CD138^+^ cells from MM patients (Figure 5d). Silencing of Sp1 by siRNAs in MM and WM cells also resulted in upregulation of miR-23b expression levels (Figure 5e).

## Discussion

Extensive gene expression profile analysis has provided interesting insight into the disease biology, and its correlation with clinical outcome is providing a new direction for risk stratification as well as novel targeted therapy. However, we have begun to realize the significant limitations of expression profile data alone and there is a growing understanding that additional genomic correlates need to be considered for an integrated oncogenomic analysis.^28^ microRNAs (miRs), a class of small non-coding RNAs targeting multiple mRNAs, are important transcriptome modifiers that alter gene function, affect the tumor cell behavior and are aberrantly expressed in myeloma.^18, 29^ miRNA-23b (miR-23b), belonging to the miR-23b–27b–24-1 cluster (9q22.32), is highly conserved in all vertebrates. Among miRNAs, it has been described as a pleiotropic modulator in different organs especially with regard to cancer development and metastasis.^30^ MiR-23b, like other miRNAs, has been found to be up- or downregulated in tumors compared with normal tissues, thus supporting its dual role in carcinogenesis as either a tumor suppressor or tumor promoter.^23^ miR-23b is downregulated in human cancers such as prostate and colon cancer, where it mediates the multiple steps of metastasis, including tumor growth, invasion and angiogenesis *in vivo.*^25, 30, 31, 32, 33^ In this study, we found miR-23b to be significantly silenced/downregulated in MM and WM as a consequence of several mechanisms.

Promoter hypermethylation-dependent silencing of tumor suppressor miRNAs has been frequently observed in hematological malignancies.^13, 34^ Here, we demonstrated for the first time that CpGs within miR-23b promoter regions are hypermethylated in MM cells and miR-23b upregulation could be achieved by the demethylating compound 5-azacytidine. Interestingly, treatment with IL-6 or supernatant from bone marrow stromal cells resulted in further decrease in miR-23b expression in MM and WM cells, indicating that the human bone marrow microenvironment has a prominent role in the modulation of miR-23b levels in myeloma cells. Moreover, miR-23b is commonly repressed in autoimmune conditions via IL-17 (ref. 24), a cytokine shown to promote myeloma cell growth.^35^ We have indeed observed further decrease in miR-23b expression in MM cells after IL-17 treatment for 24 h. The suppression of miR-23b expression in tumors and cancer cell lines suggests a tumor suppressor role in MM and WM. However, neither the functional role nor the diagnostic or prognostic implications of miR-23b in MM and WM have been previously defined. Both lentivirus- and synthetic mimics-mediated enforced expression demonstrate that miR-23b replacement causes a significant decrease in cell proliferation and survival over time *in vitro* and *in vivo*, along with induction of caspase-3/7 activity.

Dysregulation of TF features prominently in the biology of MM.^6, 36^ Transcriptional regulators and miRs appear to cooperate in the framework of a multigene transcriptional and post-transcriptional feed-forward loop.^37^ At the molecular level, we have identified Sp1, a TF endowed with oncogenic activity in MM and WM, as a target of miR-23b. On the other hand, we report here the transcriptional repression of miR-23b by c-myc, an oncogenic TF known to regulate miRs^38^ and stimulate cell proliferation.^27, 39^ Thus *MYC*-dependent miR-23b repression in myeloma cells may allow activation of oncogenic TFs Sp1 and NF-κB, representing the first feed-forward loop with critical growth and survival role in myeloma.

Taken together, these data support a model in which the humoral environment may confer a proliferative advantage partly by reducing miR-23b expression in tumor cells, suggesting a tumor suppressor role in MM and WM and highlighting the potential of a miR-23b-targeted replacement therapy to treat these hematologic malignancies.
