# Supplementary material for: miR-23b/SP1/c-myc forms a feed-forward loop supporting multiple myeloma cell growth
Source: Blood Cancer J. 2016 Jan 15;6(1):e380–. doi: 10.1038/bcj.2015.106 (PMC4742623; doi:10.1038/bcj.2015.106)
Supplement: Supplementary Table 1 [file bcj2015106x1.doc]

**Supplementary Table 1.** Sequence of the primers used for the CpG island methylation status analysis. The T7-promoter tag and the 10-mer tag sequence are underlined.

|  |  | **Primer sequences (5'-3')** | **Amplicon Length (bp)** | **Annealing temperature (C°)** |
| --- | --- | --- | --- | --- |
| CpG island 1 amplicon 1 | For | AGGAAGAGAGGAATTATGTGTGTTAGGAAAGGGAA | 486 | 62 |
|  | Rev | CAGTAATACGACTCACTATAGGGAGAAGGCTAAAAATCCCCCTACTAAAAAAAACC |  |  |
| CpG island 1 amplicon 2 | For | AGGAAGAGAGGTTAGAATTTGTTTGGAGAGAAGGG | 344 | 62 |
|  | Rev | CAGTAATACGACTCACTATAGGGAGAAGGCTAAACAACCAAAACACCTAAAAACAA |  |  |
| CpG island 2 amplicon 1 | For | AGGAAGAGAGTTGTTTTTAGGTGTTTTGGTTGTTT | 459 | 62 |
|  | Rev | CAGTAATACGACTCACTATAGGGAGAAGGCTAAATCAAATAATCTCTCATCCTCCA |  |  |
| CpG island 2 amplicon 2 | For | AGGAAGAGAGTTTGTATTTGAAGAGAAGGTGAGATG | 380 | 60 |
|  | Rev | CAGTAATACGACTCACTATAGGGAGAAGGCTCCAAAACAACAAAAACTCCTCC |  |  |
| Promoter region 1 | For | AGGAAGAGAGTTAGTTTATTGAATTTTTTTAGAGGGTA | 484 | 58 |
|  | Rev | CAGTAATACGACTCACTATAGGGAGAAGGCTAAAACAACCAAAAACAAAACAAAC |  |  |
| Promoter region 2 | For | AGGAAGAGAGTGTAATTAGGAATATTAGGGTATTAGGG | 285 | 60 |
|  | Rev | CAGTAATACGACTCACTATAGGGAGAAGGCTAACAAATTACCTATCTCAACC |  |  |
