# Supplementary figures and images for: miR-23b/SP1/c-myc forms a feed-forward loop supporting multiple myeloma cell growth
Source: Blood Cancer J. 2016 Jan 15;6(1):e380–. doi: 10.1038/bcj.2015.106 (PMC4742623; doi:10.1038/bcj.2015.106)

**Supplementary Figure 1**

**A**


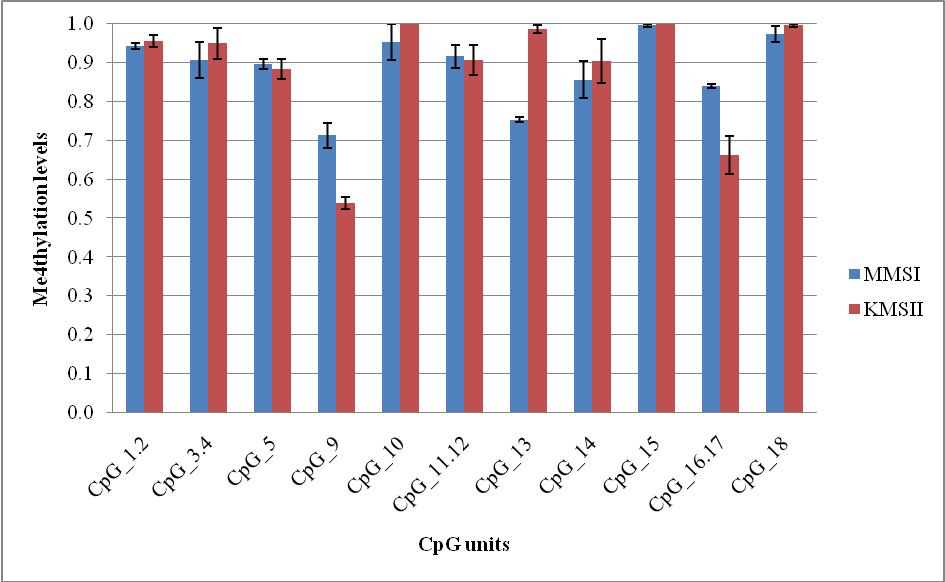


**B**


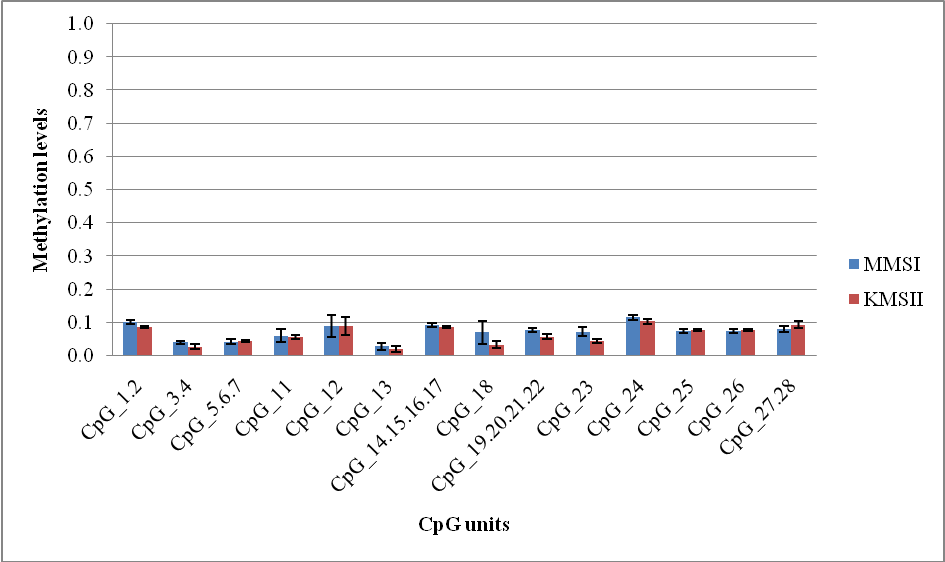


**C**

**
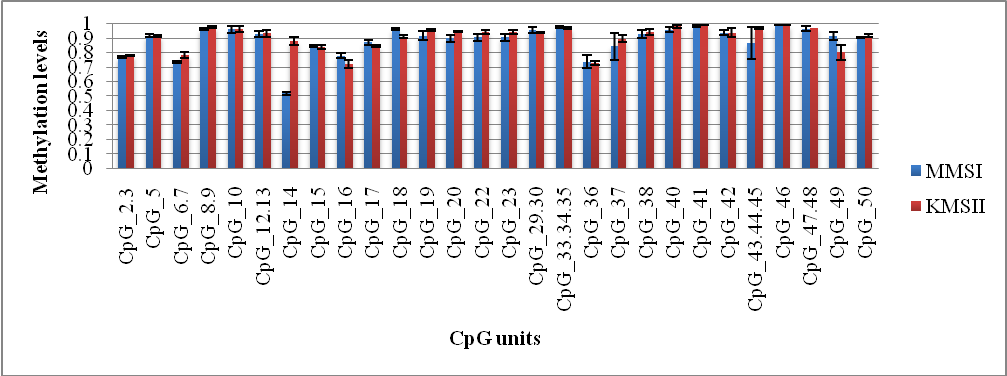
**

Supplement: Supplementary Figure 1 [file bcj2015106x2.doc]
