# Supplementary material for: miR-23b/SP1/c-myc forms a feed-forward loop supporting multiple myeloma cell growth
Source: Blood Cancer J. 2016 Jan 15;6(1):e380–. doi: 10.1038/bcj.2015.106 (PMC4742623; doi:10.1038/bcj.2015.106)
Supplement: Supplementary Figure 2 [file bcj2015106x3.doc]

**Supplementary Figure 2**

94725317 gcagctagca gggtgatgtt ataaaactag atcctgtcac ttacgtgttg ataactcttc 94725376

94725377 actagtttcc ccttacactt cgaataaact cgtattcctc acaggaaccc agagagtctg 94725436

94725437 tgcatgatgg ggactctcct tgccttcttg atctcatcct ctgttcttcc catgacctac 94725496

NF-kappaB NF-kappaB

94725497 tttctcactt tctccaaggc accacattct gccccttccc tattgcttgc tcacatgtag 94725556

NF-kappaB c-myc

94725557 attcttctct tggctgttct ttccttagct ctccacagag ttggctgctt ctcagtttac 94725616

94725617 tgaacctctt cagagggcac tttcttgaca tctcaaatca aaaggtcacc tgtcttcctt 94725676

c-myc

94725677 taatttctca gcaacttatt ttccttctac tttttttttt ttttttgaga tggagtctcg 94725736

94725737 ctgtcgccca ggttggagtg cagtggcgcg atctcggctc actgcaggct ccgccccccg 94725796

Sp1 Sp1

94725797 gggttcccgc cattctcctg cctcagcctc ccgagtagct gggactacag gcgcccacca 94725856

NF-kappaB/Sp1 Sp1 Sp

94725857 cctcgcccgg ctaatttttt gtatttttag tagagacggg gtttcaccgt gttagccagg 94725916

Sp1

94725917 atggtctcga tctcctgact ttgtgatccg cctgcctcgg cctcccaaag tgctgggatt 94725976

Sp1

94725977 acaagcgtga gccaccgcgc ccggcctttc cttctacatt taatgtttgc tttctcaact 94726036

Sp1 NF-kappaB/Sp1 Sp Sp

94726037 agaaagtaat ccccatgagg gaaggcacag cgtctgtctt gttcctggtt gtttccccag 94726096

NF-kappaB NF-kappaB

94726097 cttttgttaa gggtgcttgc ctggcacaca ggaggacctt aaattgtggt ggaattaata 94726156

94726157 tgtgaacaat agtcacatgc agcagaggtg agactaggct aaggaataaa attgcacatc 94726216

c-myc

94726217 tgtatttcag taactttttt taaattttat ttgaaacagg gtcttgctct gtggctcagg 94726276

94726277 gtggagtgta gtggcacgtt catagctcat tgcagcctcg aactcctgga cctaagcaat 94726336

94726337 cctcctgcct cagcctccat agtagctgga ccacaggtgc acgccaccac actcggctaa 94726396

c-myc

94726397 ttttttaatt tttttgtaga gacggggcct ccccaggttg cccaggctgg tgattttttt 94726456

94726457 cttaagcttc ataaagattt tgcaattagg aatactaggg taccagggca actctacaag 94726516

94726517 ccctagccag ccaagctgct cgcgccctcc tatgaccgcg gcagctccgc gcctgcgcag 94726576

Sp1

94726577 atctcccccg ggcggtctcg ccctctcatc ctaagggggc gggcctagcg gaaggcgggt 94726636

Sp1 Sp1 Sp1

94726637 caggcagaat gcggccccgg gcgcgtgtgg atgacgtcac ccggcgcgtg ccggggtagc 94726696

Sp1

94726697 ccgtagtaac cccgagtctg cggaagtggt gacccgtggg acgcggctga gacaggtaac 94726756

94726757 ctgttcgctc cctgt 94726816
