# Supplementary material for: miR-23b/SP1/c-myc forms a feed-forward loop supporting multiple myeloma cell growth
Source: Blood Cancer J. 2016 Jan 15;6(1):e380–. doi: 10.1038/bcj.2015.106 (PMC4742623; doi:10.1038/bcj.2015.106)
Supplement: Supplementary Information [file bcj2015106x4.doc]

**Supplementary Table 1.** Sequence of the primers used for CpG island methylation analysis. The T7-promoter tag and the 10-mer tag sequence are underlined.

**Supplementary Figure 1.** Mean methylation levels of CpG sites in *miR-23b* promoter region 1 (A) and 2 (B) and in CpG islands (C), as described in Results, according to Sequenom MassARRAY analysis. Data are expressed as mean ±SD of 3 independent experiments performed in triplicate.

**Supplementary Figure 2**. Bioinformatic analysis of TF- binding sites within miR-23b promoter by TRANSFAC database.
